# Two-year outcomes of Micra AV leadless pacemakers in the Micra AV CED study

**DOI:** 10.1093/europace/euae273

**Published:** 2024-11-01

**Authors:** Mikhael F El-Chami, Lucas Higuera, Colleen Longacre, Kurt Stromberg, George Crossley, Jonathan P Piccini

**Affiliations:** Department of Medicine, Division of Cardiology, Emory University School of Medicine, 100 Woodruff Circle, 550 Peachtree Street NE, Atlanta, GA 30308, USA; Medtronic, Inc., Minneapolis, MN, USA; Medtronic, Inc., Minneapolis, MN, USA; Medtronic, Inc., Minneapolis, MN, USA; Department of Medicine, Vanderbilt University Medical Center, Nashville, TN, USA; Department of Medicine, Duke University School of Medicine, Durham, NC, USA

**Keywords:** Leadless pacemakers, Transvenous pacemakers, System re-intervention, Complications, Survival

## Abstract

**Aims:**

Leadless pacing is a safe and effective alternative to transvenous pacing for bradycardia. Micra AV is a leadless, single-device solution that provides atrioventricular synchronous ventricular pacing therapy. Early results from the Micra AV CED study showed reductions in short-term complications associated with the Micra AV leadless pacemaker among US Medicare patients. The objective of this study is to compare chronic complications, re-interventions, and all-cause mortality at 2 years between patients implanted with a Micra AV leadless pacemaker and a traditional dual-chamber transvenous (DC-TV) pacemaker.

**Methods and results:**

Patients implanted with a Micra AV leadless pacemaker (*n* = 7552) or a DC-TV pacemaker (*n* = 110 558) in 2020 and 2021 were identified using device registration-linked Medicare administrative claims data. Competing risk models compared the unadjusted and propensity score overlap weight–adjusted complication, re-intervention, and all-cause mortality rates of Micra AV and DC-TV patients at 2 years. Micra AV patients had significantly more comorbidities (end-stage renal disease 14.9 vs. 2.0%, *P* < 0.0001; renal dysfunction 47.9 vs. 34.2%, *P* < 0.0001; diabetes 46.2 vs. 38.3%, *P* < 0.001; congestive heart failure 41.4 vs. 30.6%, *P* < 0.0001). Two years post-implant, Micra AV patients had lower complication rates [adjusted 5.3 vs. 9.6%, hazard ratio (HR): 0.54, 95% confidence interval (CI) 0.49–0.61, *P* < 0.0001] and lower re-intervention rates (adjusted 3.5 vs. 5.6%, HR: 0.62, 95% CI 0.54–0.72, *P* < 0.0001) than DC-TV patients. Upgrades to cardiac resynchronization therapy were low in both groups (adjusted 1.6 vs. 1.7%, *P* = 0.40), as were Micra AV upgrades to a dual-chamber system (adjusted 1.4%). All-cause mortality rates remained higher in Micra AV than in DC-TV patients (unadjusted HR: 2.48, 95% CI 2.35–2.62, *P* < 0.0001; adjusted HR: 1.53, 95% CI 1.44–1.62, *P* < 0.0001).

**Conclusion:**

Patients implanted with Micra AV had lower complications and re-intervention rates at 2 years than patients implanted with a traditional DC-TV pacemaker. All-cause mortality remained higher in Micra AV patients, likely due to their higher comorbidity burden and other differences in baseline characteristics.

**Clinical trial registration:**

ClinicalTrials.gov ID NCT04235491

## Graphical Abstract

**Graphical Abstract:**
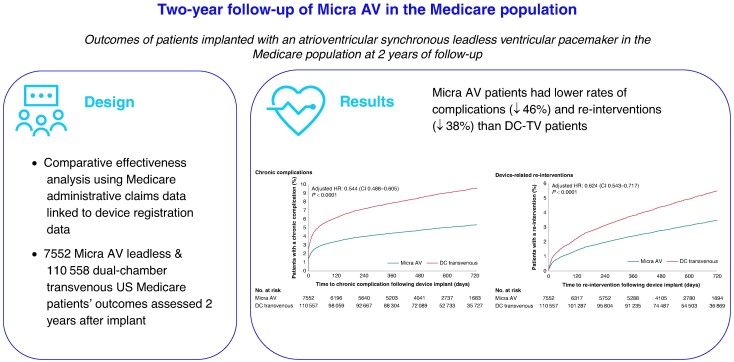

**What’s new?**

- Micra AV is a leadless pacemaker that provides atrioventricular (AV) synchrony for patients with AV block in a single-device solution.
- Compared with patients with dual-chamber transvenous (DC-TV) pacemakers, patients implanted with Micra AV have 48% fewer complications and 38% fewer re-interventions 2 years after implant.
- Upgrade rates to cardiac resynchronization therapy devices are low and not statistically different between Micra AV and DC-TV pacemaker patients.

## Introduction

Leadless pacemakers are intra-cardiac devices that provide pacing for bradyarrhythmias without the need for transvenous leads. While some leadless pacemakers consist of two devices implanted in the right atrium and right ventricle, the Micra AV pacemaker is the only market-released leadless pacemaker that provides a single-device solution with ventricular accelerometer-based atrioventricular (AV) synchronous pacing for patients with AV block (AVB).^1^ Previous studies have shown that Micra VR, a leadless VVI pacemaker, is associated with fewer complications and device-related re-interventions than single-chamber transvenous pacemakers.^1–5^

The Center for Medicare and Medicaid Services (CMS) issued a National Coverage Determination for Micra AV in March 2020, which requires a Coverage with Evidence Development (CED) study including all Medicare beneficiaries receiving a Micra AV leadless pacemaker (NCT03039712). The coverage determination requires publication of pre-specified 30-day acute complications and 2-year chronic complications and device-related interventions. Earlier results from the Micra AV CED study showed that, compared with patients with dual-chamber transvenous (DC-TV) pacemakers, patients with Micra AV have lower rates of complications 30 days after implant and lower rates of chronic complications and device-related re-interventions 6 months after implant.^6^ There is no published contemporaneous comparative evidence of Micra AV for long-term safety and efficacy; this study aims to compare pre-specified chronic complications, device-related re-interventions, and all-cause mortality of Micra AV patients compared with patients with a DC-TV pacemaker 2 years after implant.

## Methods

The Longitudinal Coverage With Evidence Development Study on Micra AV Leadless Pacemakers (Micra AV CED) study (NCT 04235491) has been described previously^6^ and follows the structure of the Micra VR CED study.^3,7^ The Micra AV CED study is a prospective, continuously enrolling study designed to evaluate complications and outcomes of the Micra AV leadless pacing system in the US Medicare population. The study uses Medicare Fee-for-Services (FFS) administrative claims data linked to Medtronic’s device registration data (DTRAK) to enrol patients, ascertain patient characteristics, identify comorbidities, and measure outcomes. Patients with Micra AV pacemakers were compared with a contemporaneous control group of patients receiving a DC-TV pacemaker, identified in the claims data. The study was approved by the Western Institutional Review Board with a waiver of informed consent and is registered on ClinicalTrials.gov (NCT04235491).

Medicare FFS claims data were used to identify beneficiaries implanted from 5 February 2020 to 31 December 2021 with either a leadless or a DC-TV pacemaker, using procedure codes in the International Classification of Diseases, 10th Revision, Procedure Coding System and the Current Procedural Terminology for implants occurring in the inpatient hospital setting or the outpatient hospital setting, respectively (see Supplementary material online, *Table S1*). The implant date was considered the index date. DTRAK information was used to identify Micra AV pacemaker implants (Model MC1AVR1, Medtronic, Inc.) from the leadless pacemaker implanted population.^7^ Dual-chamber transvenous patients implanted in a hospital or clinic without Micra AV patients were excluded. Patients with evidence of a prior cardiovascular implantable electronic device or without at least 12 months of pre-implant continuous enrolment in Medicare FFS were also excluded.

Patient comorbidities and baseline patient characteristics were assessed 12 months before the index date using diagnosis and procedure codes present on any encounter (see Supplementary material online, *Table S2*) using Medicare claims and enrolment data. Implant encounter characteristics (inpatient or outpatient hospital setting, admission through an emergency department, admission during the weekend, concomitant cardiac ablation or transcatheter aortic valve procedures, and the number of days from hospital admission to implant procedure) were also measured. The Charlson Comorbidity Index was calculated for each patient to assess the overall patient acuity.^8^

Pre-specified outcomes [chronic complications: embolism, thrombosis, device-related complications, pericarditis, and haemothorax; device-related re-interventions: system revision, lead revision or replacement, system replacement, system removal, leadless-to-transvenous or transvenous-to-leadless replacement, or upgrade to a cardiac resynchronization therapy (CRT) device; and all-cause mortality] were measured up to 2 years after the index date, following the diagnosis and procedure codes described in Supplementary material online, *Tables S1* and *S2*. These complication measures were defined in the Micra CED study protocol and approved by the Centers for Medicare and Medicaid Services to fulfil its CED requirement. Several prior publications of the Micra CED study have used these definitions of complications.^2–6^ This study does not have a clinical adjudication committee; therefore, it is not possible to determine whether complications had a significant clinical impact, such as a prolonged hospitalization. Thus, the measured event rates in the CED studies are known to be higher than those in clinical studies, as the definition is more comprehensive. However, Wherry *et al*.^7^ showed that claims data accurately identified clinical events/complications in patients implanted with leadless pacemakers. In the case of over- or misspecification of events, this will occur at both the Micra AV and the DC-TV patients and thus should not affect the differential in event rates.

### Statistical analysis

Fine–Gray competing risk models with all-cause death as a competing risk were used to compare 2-year chronic complications and device-related re-interventions and chronic complications, and Cox proportional hazard models were used to compare 2-year all-cause mortality. Propensity score overlap weights based on the propensity of a given patient to be implanted with a Micra AV were used to adjust the results for patient characteristics.^9,10^ Model standard errors were correlated at the hospital level to account for within-hospital correlation. Events occurring between 1 and 10 patients were suppressed to protect beneficiary privacy as required by CMS.^11^ All statistical analyses were conducted in SAS version 9.4 (SAS Institute).

Three sensitivity analyses were included. First, an endpoint of 2-year all-cause mortality conditional on 6-month survival was included to separate any effects of differences in patient acuity reflected on early mortality from late mortality better attributed to the device. The rationale of this analysis is that patients who survived an initial period are more similar in patient acuity than the overall cohort. Second, a falsification analysis using hip fracture as an endpoint was included to test for residual confounding; under the assumption of no correlation between pacemaker choice and hip fractures, any differences between Micra AV and DC-TV patients in this outcome indicate the presence of residual confounding. Lastly, a subgroup analysis of all-cause mortality in AVB patients identified using a claims-based algorithm^12^ was included to refine the comparison in patients with the standard indication for Micra AV pacemakers.

## Results

There were 7552 Micra AV and 110 558 DC-TV *de novo* implant procedures performed in 1244 unique providers identified in the data (see Supplementary material online, *Figure S1*). Patient baseline characteristics are detailed in *Table 1*. In general, Micra AV patients were more likely to have any comorbidities than DC-TV patients, particularly end-stage renal disease (ESRD) (14.9 vs. 2.0%, *P* < 0.0001), renal dysfunction (47.9 vs. 34.2%, *P* < 0.0001), diabetes (46.2 vs. 38.3%, *P* < 0.0001), and congestive heart failure (41.4 vs. 30.6%, *P* < 0.0001), and have a higher mean Charlson Comorbidity Index score (5.0 ± 3.4 vs. 3.9 ± 3.0, *P* < 0.0001). Compared with DC-TV implant procedures, Micra AV implant procedures were more likely to be inpatient (68.6 vs. 53.1%, *P* < 0.0001), to originate from the emergency department (15.5 vs. 10.5%, *P* < 0.0001), and to have used temporary pacing and life-saving procedures during the implant hospitalization (14.6 vs. 7.6%, *P* < 0.0001; 34.5 vs. 20.7%, *P* < 0.0001, respectively).

**Table 1:** Baseline characteristics

	Micra AV	DC-TV	*P*-value
***n***	7552	110 558	
**Patient characteristic**			
Age at implant, mean ± SD (range)	79.0 ± 10.2 (21–105)	78.7 ± 8.0 (23–106)	0.0146
Female	3635 (48.1%)	51 650 (46.7%)	0.0171
**Patient comorbidity**			
ESRD	1126 (14.9%)	2191 (2.0%)	<0.0001
Renal dysfunction	3621 (47.9%)	37 852 (34.2%)	<0.0001
Coronary artery disease	3750 (49.7%)	53 761 (48.6%)	0.0835
Peripheral vascular disease	1927 (25.5%)	21 824 (19.7%)	<0.0001
Tricuspid valve disease	1572 (20.8%)	21 971 (19.9%)	0.0472
Atrial fibrillation	3050 (40.4%)	49.823 (45.1%)	<0.0001
Left bundle branch block	682 (9.0%)	8056 (7.3%)	<0.0001
Supraventricular tachycardia	624 (8.3%)	12 164 (11.0%)	<0.0001
Ventricular arrhythmia	1043 (13.8%)	18 034 (16.3%)	<0.0001
Prior acute myocardial infarction	1274 (16.9%)	15 494 (14.0%)	<0.0001
Prior coronary artery bypass graft	796 (10.5%)	12 515 (11.3%)	0.0382
Prior TAVR	206 (2.7%)	1910 (1.7%)	<0.0001
Prior percutaneous coronary intervention	1023 (13.5%)	15 339 (13.9%)	0.4246
Diabetes	3487 (46.2%)	42 323 (38.3%)	<0.0001
Congestive heart failure	3127 (41.4%)	33 784 (30.6%)	<0.0001
Chronic obstructive pulmonary disease	1867 (24.7%)	22 882 (20.7%)	<0.0001
Hyperlipidaemia	5598 (74.1%)	84 927 (76.8%)	<0.0001
Hypertension	6778 (89.8%)	99 075 (89.6%)	0.7047
COVID-19	631 (8.4%)	5855 (5.3%)	<0.0001
Charlson Comorbidity Index, mean ± SD (range)	5.0 ± 3.4 (0–20)	3.9 ± 3.0 (0–21)	<0.0001
**Implant encounter characteristic**			
Inpatient implant	5177 (68.6%)	58 740 (53.1%)	<0.0001
Weekend implant	295 (3.9%)	4138 (3.7%)	0.4697
Emergency admission	1171 (15.5%)	11 577 (10.5%)	<0.0001
Concomitant atrial ablation	445 (5.9%)	2404 (2.2%)	<0.0001
Concomitant TAVR	391 (5.2%)	3645 (3.3%)	<0.0001
Use of temporary pacing during implant hospitalization	1100 (14.6%)	8432 (7.6%)	<0.0001
Use of life-saving procedures during implant hospitalization	2603 (34.5%)	22 832 (20.7%)	<0.0001
**AV block indication^a^**	**5607 (74.2%)**	**52 652 (47.6%)**	**<0**.**0001**

Concomitant procedures occurred during the same encounter as the pacemaker implantation. Live-saving procedures consist of cardiopulmonary resuscitation, use of extracorporeal membrane oxygenation/life support, use of critical care services, and use of ventilator and ventilation assistance.

AV, atrioventricular; DC-TV, dual-chamber transvenous pacemaker; ESRD, end-stage renal disease; SD, standard deviation; TAVR, transcatheter aortic valve replacement.

^a^Result of indication algorithm published in Tonegawa *et al*.^12^

**Figure 1:**
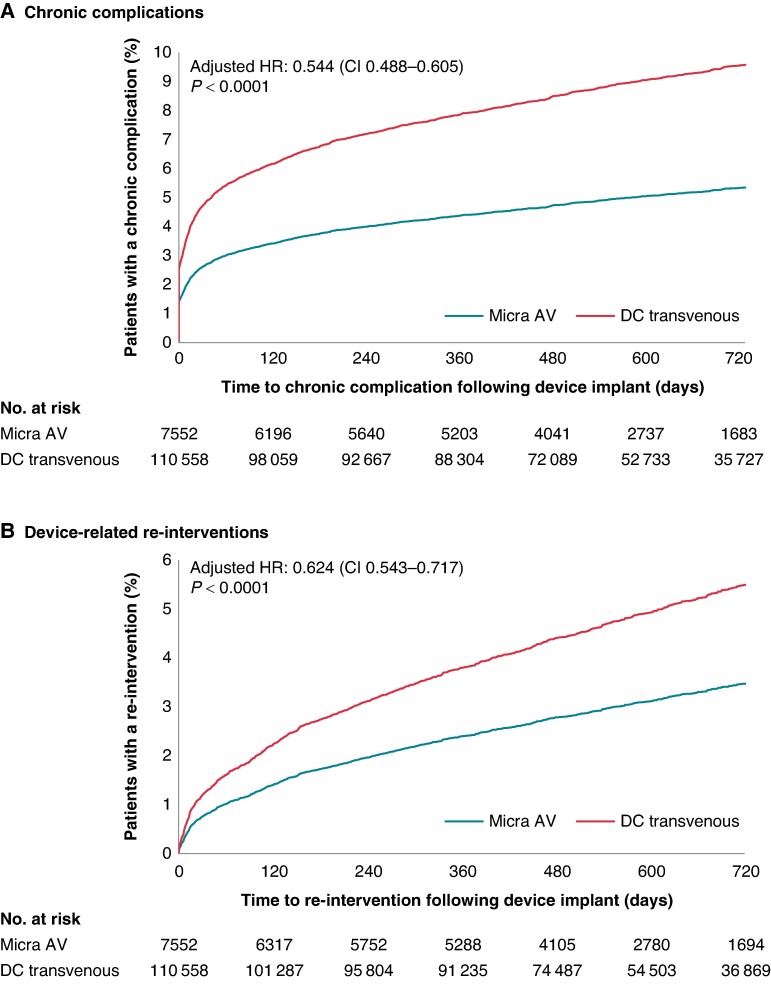
Cumulative incidence functions of chronic complications and device-related re-interventions at 2 years in Micra AV vs. dual-chamber transvenous patients. Adjusted HRs, 95% CIs, and cumulative incidence functions for 2-year chronic complications (*A*) and device-related re-interventions (*B*) based on Fine–Gray competing risk models. CI, confidence interval; DC, dual-chamber; HR, hazard ratio.

*Figure 1* shows the estimated cumulative incidence function (CIF) of the adjusted Fine–Gray models that compare chronic complications (Figure 1*A*) and device-related re-interventions (*Figure 1B*) between Micra AV and DC-TV patients. Through 2 years, Micra AV patients had 46% fewer chronic complications [adjusted hazard ratio (HR): 0.544, 95% confidence interval (CI): 0.488–0.605] and 38% fewer device-related re-interventions (adjusted HR: 0.624, 95% CI: 0.543–0.717) than DC-TV patients. *Table 2* shows the comparison of the components of the chronic complication and re-intervention measures. Overall, the difference in chronic complications was driven by the significantly lower rate of device-related complications in Micra AV patients vs. DC-TV patients at 2 years (2.9 vs. 6.8%, *P* < 0.0001). There were no statistical differences in embolism and thrombosis (0.2 vs. 0.2%, *P* = 0.9015), pericarditis (1.7 vs. 1.8%, *P* = 0.6876), or haemothorax (0.7 vs. 0.7%, *P* = 0.7931). Among device-related re-interventions, Micra AV patients had fewer revisions and removals, but no statistical differences in replacements (0.5 vs. 0.6%, *P* = 0.3356) or upgrades to CRT devices (1.6 vs. 1.7%, *P* = 0.3955). The replacement rate from Micra AV to DC-TV was 1.4% (adjusted rate = 1.4%, 95% CI: 1.2–1.8%) while the replacement rate from DC-TV to Micra was 0.2% (adjusted rate = 0.2%, 95% CI: 0.2–0.3%). These events were included in the overall re-intervention rate, but not statistically compared, as the need for upgrade is not symmetrical: for instance, dual-chamber pacemakers can deliver atrial pacing.

**Table 2:** Adjusted rates of chronic complications, device-related re-interventions, and all-cause mortality at 2 years in Micra AV vs. dual-chamber transvenous patients

	Micra AV (*n* = 7552)	DC-TV (*n* = 110 558)	Micra AV vs. DC-TV	
	2-year weighted CIF estimates (95% CI)	2-year weighted CIF estimates (95% CI)	Relative risk reduction (95% CI)	*P*-value
**Overall complications**	**5.3%** (**5.1–5.5%)**	**9.6%** (**9.3–9.9%)**	**46%** (**40–51%)**	**<0**.**0001**
Embolism and thrombosis	0.2% (0.2–0.2%)	0.2% (0.2–0.2%)	3% (−64 to 43%)	0.9015
Device-related complications	2.9% (2.8–2.9%)	6.8% (6.7–6.9%)	59% (53–64%)	<0.0001
Breakdown	1.8% (1.6–1.9%)	3.0% (2.8–3.2%)	41% (29–51%)	<0.0001
Dislodgement	0.5% (0.5–0.5%)	2.8% (2.7–2.9%)	83% (76–88%)	<0.0001
Other mechanical failure	0.8% (0.7–0.8%)	1.5% (1.3–1.6%)	48% (30–61%)	<0.0001
Infection	^a^	0.6% (0.5–0.6%)	96% (83–99%)	<0.0001
Device pain	^a^	0.4% (0.4–0.5%)	74% (48–87%)	0.0002
Device stenosis	0.5% (0.4–0.6%)	0.6% (0.5–0.7%)	14% (−23 to 40%)	0.4152
Pocket complications	N/A	1.6% (1.5–1.7%)	NE	NE
Other complications	2.1% (2.0–2.1%)	2.0% (2.0–2.1%)	−2% (−22 to 14%)	0.7873
Pericarditis	1.7% (1.5–1.8%)	1.8% (1.6–1.9%)	4% (−18 to 22%)	0.6876
Haemothorax	0.7% (0.6–0.8%)	0.7% (0.6–0.8%)	4% (−30 to 29%)	0.7931
**Overall re-interventions**	**3.5%** (**3.3–3.7%)**	**5.6%** (**5.2–5.9%)**	**38%** (**28–46%)**	**<0**.**0001**
Revisions	^a^	1.5% (1.4–1.6%)	94% (88–97%)	<0.0001
Lead-related re-interventions	N/A	1.3% (1.2–1.4%)	NE	NE
Replacement	0.5% (0.4–0.6%)	0.6% (0.6–0.7%)	22% (−29 to 53%)	0.3356
Micra AV upgrades to DC-TV	1.4% (1.2–1.8%)	N/A	N/A	N/A
DC-TV upgrades to Micra AV	N/A	0.2% (0.2–0.3%)	N/A	N/A
Removal	^a^	0.7% (0.6–0.8%)	83% (66–91%)	<0.0001
Upgrade to CRT	1.6% (1.4–1.7%)	1.7% (1.6–1.9%)	9% (−13 to 27%)	0.3955
**All-cause mortality**	**34.0%** (**33.3–34.7%)**	**23.8%** (**23.2–24.4%)**	**−53%** (**−62 to 44%)**	**<0**.**0001**

AV, atrioventricular; CI, confidence interval; CIF, cumulative incidence function; CRT, cardiac resynchronization therapy; DC-TV, dual-chamber transvenous pacemaker; N/A, not applicable; NE, not estimable.

^a^Cell value between 1 and 10.

**Figure 2:**
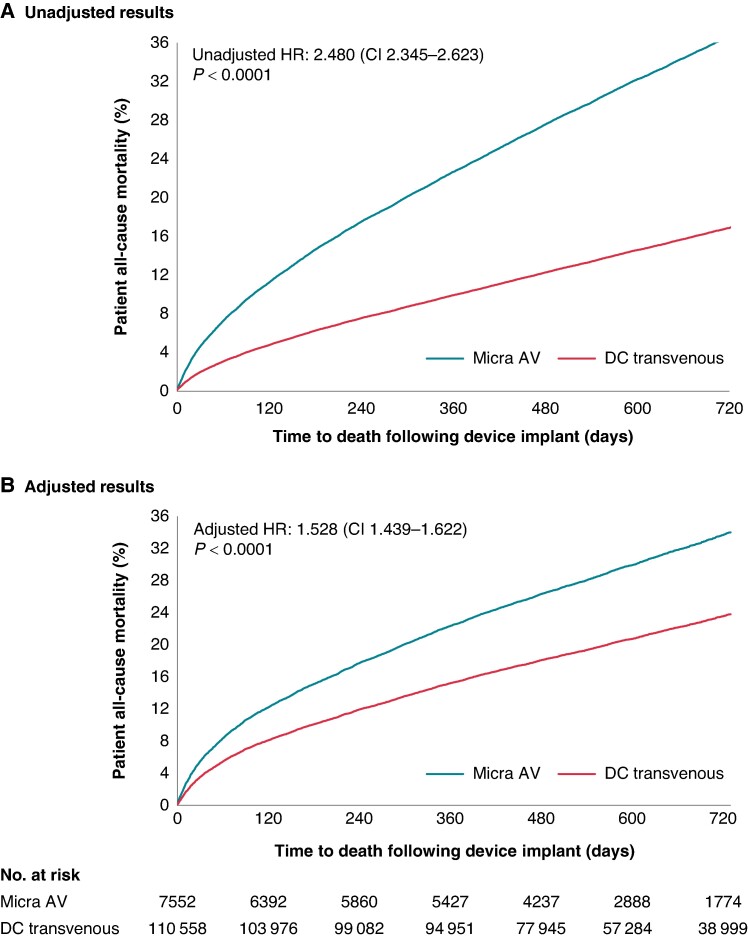
Cumulative incidence functions of all-cause mortality at 2 years in Micra AV vs. dual-chamber transvenous patients. Unadjusted (*A*) and adjusted (*B*) HRs, 95% CIs, and cumulative incidence functions for 2-year all-cause mortality based on Fine–Gray competing risk models. CI, confidence interval; DC, dual-chamber; HR, hazard ratio.

*Figure 2* shows the unadjusted (*Figure 2A*) and adjusted (*Figure 2B*) CIFs of all-cause mortality in Micra AV and DC-TV patients. Micra AV patients had a higher unadjusted all-cause mortality rate than DC-TV patients (unadjusted HR: 2.480, 95% CI: 2.345–2.623); adjusting for measured patient and encounter characteristics reduced the magnitude of the difference but did not eliminate it (adjusted HR: 1.528, 95% CI: 1.439–1.622). Adjusted event rates are reported in *Table 2*, unadjusted event rates are reported in Supplementary material online, *Table S3*, and unadjusted CIFs and HR are shown in Supplementary material online, *Figure S2*.

The sensitivity analyses are reported in Supplementary material online, *Appendix S1*. In the falsification test, Micra AV patients had higher hip fracture rates than DC-TV patients at 2 years (adjusted HR: 1.275, 95% CI: 1.028–1.581; Supplementary material online, *Table S4*). Conditional on survival 6 months after implant, the differential in all-cause mortality rate was lower but still statistically significant (unadjusted HR: 2.093, 95% CI: 1.953–2.243 and adjusted HR: 1.392, 95% CI: 1.295–1.495; Supplementary material online, *Figure S3*). In our cohort, 5607 Micra AV and 52 652 DC-TV patients were identified as AVB patients in the data; all-cause mortality was higher in Micra AV patients than in DC-TV patients in this AVB cohort (adjusted HR: 1.509, 95% CI: 1.414–1.611; Supplementary material online, *Table S5*), replicating the results from the overall cohort.

## Discussion

At 2 years of follow-up, the Micra AV CED study shows that compared with patients implanted with dual-chamber pacemakers, patients with a Micra AV pacemaker have 46% fewer chronic complications and 38% fewer device-related re-interventions. These results are consistent with previous analyses of ventricular leadless pacemakers vs. single-chamber ventricular transvenous pacemakers: 31 and 32% fewer chronic complications and 38 and 41% fewer re-interventions with the Micra VR leadless VVI pacemaker at 2 years^2^ and 3 years,^4^ respectively, which is not surprising given the lower likelihood of device complications in single-chamber transvenous pacemakers compared with DC-TV pacemakers. These results are also in line with the 6-month follow-up results of the Micra AV CED study (50% fewer chronic complications and 54% fewer re-interventions). In addition, these results corroborate those from the Micra AV post-approval registry: a 3.7% post-implant complication rate at 12 months in Micra AV patients, compared with an 8.8% rate from a historical comparison of DC-TV patients.^13^ Micra AV patients have lower device-related complications, revisions, and removals, but there are no differences in upgrades to CRT devices. All-cause mortality is higher in Micra AV patients, likely due to more comorbidities and differences in patient characteristics in this population.

The 6-month follow-up results of the Micra AV CED study appeared to be affected by residual confounding due to unmeasured differences in the treatment groups.^6^ In particular, Micra AV patients seemed to be sicker at implant in ways that the statistical adjustments based on comorbidity and encounter characteristics measurable in claims could not account for, which resulted in unadjusted and adjusted all-cause mortality rates higher than in DC-TV patients. The sensitivity analyses undertaken in that publication showed that patient mortality *among patients implanted with a DC-TV* increased significantly as their propensity to have been implanted with Micra increased: among patients who received a DC-TV, those in the highest quintile of the propensity of receiving a Micra AV have over six times higher all-cause mortality at 6 months than those in the lowest quintile of that propensity (pages 70–71).^6^ In this study, we take advantage of the longer follow-up period to analyse whether differences in all-cause mortality rates diminish when the initial effect of acuity has waned, that is, when implanted patients have survived an initial period. Conditional on survival 6 months after implant, the differential in all-cause mortality rates between Micra AV and DC-TV patients was lower than the unconditional analysis; however, it was still higher in Micra AV patients. The updated falsification endpoint analysis also suggests the presence of residual confounding in this study: Micra AV patients had a higher rate of a negative outcome (hip fracture) unrelated to the choice of transvenous or leadless pacemaker but related to the higher patient acuity of Micra AV patients.

A reassuring finding in this study is the low rate of Micra AV upgrade to DC-TV pacemakers (1.4%). This is in line with the 1-year results of the Micra AV PAR that reported three patients (0.38%) with pacemaker syndrome requiring an upgrade to DC-TV pacemaker (two patients) or CRT (one patient). These results suggest that the Micra AV, a VDD pacemaker that provides a mean AV synchrony of 80–84%,^13,14^ is clinically well tolerated in most patients who otherwise would have been implanted with a DC-TV pacemaker. Furthermore, upgrades to a CRT system were similarly low in both groups (1.6–1.7%) in a population where between 30.6% (DC-TV patients) and 41.4% (Micra AV patients) have a prior diagnosis of congestive heart failure. Cardiac resynchronization therapy upgrades were also uncommon in other leadless VR and AV studies. The 5-year follow-up of the Micra VR PAR reported a 2% rate of CRT upgrades.^15^ Similarly, only five patients (5/796) required CRT upgrades in the Micra AV PAR over a 1-year follow-up.^13^ This low upgrade rate is encouraging and suggests that pacing-induced cardiomyopathy is uncommon in this group of patients and that the imperfect AV synchrony algorithm does not increase the need for CRT requirements.

Overall, the results of this study suggest that Micra AV is a reasonable alternative to DC-TV pacemakers in the selected group of patients. The advantage of this technology over the traditional DC-TV pacemaker, which includes reducing intermediate and long-term complications predominantly driven by lower rates of device-related complications and re-interventions, has been shown in all the Micra studies.^2,4,15^ Despite the higher comorbidity burden in Micra AV patients compared with DC-TV patients, there was a reduction in complications and re-interventions associated with Micra AV implants. The main drawback of this technology is the higher rate of periprocedural perforations compared with DC-TV pacemakers.^16^ Recently, a dual-chamber leadless pacemaker was approved by the Food and Drug Administration. While this device would benefit patients with sinus node dysfunction, Micra AV might still be desired in patients with intact sinus node function and conduction system disease for two main reasons: first, to reduce the number of leadless devices in the heart, thus possibly minimizing complications, and second, the use of dual-chamber leadless pacemakers may result in a significant impact on battery longevity due to device-to-device communication. On the other hand, adequate AV synchrony achieved through mechanical atrial detection requires additional involvement from the physician and can be enhanced through optimal programming strategies^17^; therefore, access to a knowledgeable team including physicians, device engineers, and arrhythmia clinic nurses can be relevant to the efficacy of Micra AV. Therefore, patient selection is important when choosing the appropriate device for the appropriate patient. Patients should also be informed about the pros and cons of each available technology and be engaged in a shared decision-making to help make the final decision about the type of device to be implanted.^18^

### Study limitations

This study shares the limitations of the previous Micra CED publications. Outcomes could be inadequately measured in administrative claims, but this is unlikely and applies to both treatment groups.^7^ More specific information about outcomes, such as reasons for revisions, upgrades, or replacements (battery depletion, device malfunction, upgrade to conduction system pacing therapy, etc.), is not available in the claims data. Claims data do not capture other relevant parameters for this population, such as pacing indication, AV synchrony, left ventricular ejection fraction, or pacing percentage, which could be relevant in understanding the differences in outcomes between Micra AV and DC-TV patients. The population under study is limited to Medicare fee-for-service enrolees (patients ≥ 65 years, disabled, or with ESRD); however, previous evidence shows that the safety benefits from Micra pacemakers extend to patients enrolled in Medicare Advantage plans (commercial managed-care plans for Medicare-eligible patients).^19^ Lastly, even after adjusting for patient and encounter characteristics, there is evidence of residual confounding in this study. The lack of patient frailty information is a potential source of residual confounding.^20^ However, as the bias from residual confounding comes from Micra AV patients being sicker, the results of this study may understate the comparative safety benefits of the Micra AV pacemaker.

## Conclusions

In this Micra AV CED study update, the Micra AV leadless pacemaker was associated with a 46% lower rate of complications and a 38% lower rate of re-interventions at 2 years compared with DC-TV pacemakers. Micra AV patients have lower device-related complications, revisions, and removals but no differences in upgrades to CRT devices. The higher all-cause mortality rate in Micra AV patients is likely due to more comorbidities and differences in patient characteristics.

## Supplementary Material

euae273_Supplementary_Data

## Data Availability

The authors are not owners of the data set (data set is owned by the Centers for Medicare and Medicaid Services) and do not have the right to share the data.
