# Supplementary material for: Two-year outcomes of Micra AV leadless pacemakers in the Micra AV CED study
Source: Europace. 2024 Nov 1;26(11):euae273. doi: 10.1093/europace/euae273 (PMC11558452; doi:10.1093/europace/euae273)
Supplement: euae273_Supplementary_Data [file euae273_supplementary_data.docx]

**Supplemental Appendices**

**Supplementary Table T1. Current Definitions of Procedural Terminology for Implants**

| **Procedure**^1^ | **CPT Codes** | **ICD-10-PCS Codes** |
| --- | --- | --- |
| **MICRA** |  |  |
| Insertion | 0387T or 33274 | 02HK3NZ *without* 02PA3NZ |
| Replacement | NA | 02HK3NZ *and* 02PA3NZ |
| Revision (e.g., repositioning) | 33999 | 02WA3NZ |
| Removal | 0388T or 33275 | 02PA3NZ *without* 02HK3NZ |
| **PACEMAKER (CONVENTIONAL), SINGLE CHAMBER with SINGLE TRANSVENOUS RIGHT VENTRICULAR LEAD** | | |
| Insertion of whole system | 33207 *without* (33222 *and* 33215 *and* 33218 *and* 33227 *and* 33228 *and* 33213 *and* 33208 *and* 33214 *and* 33999 *and* 33233 *and* 33234 *and* 33225 *and* 33224 *and* 33216 *and* 33235 *and* 33224) | ( (0JH604Z *or* 0JH605Z) *and* 02HK3JZ) *without* (02PA3MZ *and* 0JPT0PZ *and* 0JPT3PZ *and* 0JH606Z *and* 02H63JZ *and* 0JH636Z *and* 0JPT3PZ *and* 02PA0MZ *and* 02PA4MZ *and* 02PAXMZ *and* 02H60JZ *and* 02H70JZ *and* 02H73JZ *and* 02HK0JZ *and* 02WA3MZ *and* 0JWT0PZ) |
| Replacement of whole system | (33207 *and* 33233 *and* 33234) *without* (33206 *and* 33225 *and* 33240 *and* 33249) | ((0JH604Z *or* 0JH605Z) *and* 02HK3JZ *and* 0JPT0PZ *and* 02PA3MZ) *without* (0JH606Z *and* 0JH607Z *and* 02H63JZ *and* 02H43JZ) |
| Insertion of generator only | 33212 *without* (33233 *and* 33216) | (0JH604Z *or* 0JH605Z) *without* (0JPT0PZ *and* 02HK3JZ *and* 02PA3MZ) |
| Replacement of generator only | ( 33227 *without* (33224 *and* 33225 *and* 33228 *and* 33213 *and* 33208 *and* 33214) ) *or* ( (33207 *and* 33233) *without* (33206 *and* 33225 *and* 33240 *and* 33249 *and* 33234) ) | ( (0JH604Z *or* 0JH605Z) *and* 0JPT0PZ ) *without* (02HK3JZ *and* 02PA3MZ *and* 0JH606Z *and* 02H63JZ *and* 02H43JZ *and* 0JH607Z) |
| Removal of whole system | (33233 *and* 33234) *without* (33216 *and* 33207 *and* 33225 *and* 33206 *and* 33240 *and* 33249) | (0JPT0PZ *and* 02PA3MZ) *without* (02HK3JZ *and* 02H63JZ *and* 02H43JZ *and* 0JH604Z *and* 0JH605Z *and* 0JH606Z *and* 0JH607Z) |
| **PACEMAKER (CONVENTIONAL), DUAL CHAMBER with TRANSVENOUS RIGHT ATRIAL AND RIGHT VENTRICULAR LEAD** | | |
| Insertion of whole system | 33208 *without* (33225 *and* 33233) | (0JH606Z *and* 02H63JZ *and* 02HK3JZ) *without* (0JPT0PZ *and* 0JPT3PZ *and* 02PA0MZ *and* 02PA3MZ *and* 02PA4MZ *and* 02PAXMZ) |
| Upgrade from single chamber to dual chamber pacemaker system | 33214 | 0JH606Z *and* 02H63JZ *and* 0JPT0PZ |
| Replacement of whole system | (33208 *and* 33233 *and* 33235) *without* (33225 *and* 33240 *and* 33249) | (0JH606Z *or* 0JH636Z) *and* 02H63JZ *and* 02HK3JZ *and* (0JPT0PZ *or* 0JPT3PZ) *and* (02PA0MZ or 02PA3MZ or 02PA4MZ or 02PAXMZ) |
| Insertion of generator only | 33213 *without* (33233 *and* 33217) | (0JH606Z *or* 0JH636Z *or* 0JH836Z *or* 0JH806Z) *without* (0JPT0PZ *and* 0JPT3PZ *and* 02H63JZ *and* 02HK3JZ *and* 02PA0MZ *or* 02PA3MZ *and* 02PA4MZ *and* 02PAXMZ) |
| Replacement of generator only | (33228 *without* (33217 *and* 33234 *and* 33235 *and* 33225) ) *or* ( (33208 *and* 33233) *without* (33225 *and* 33240 *and* 33249 *and* 33235) ) | ( (0JH606Z or 0JH636Z) *and* (0JPT0PZ *or* 0JPT3PZ) ) *without* (02H63JZ *and* 02HK3JZ *and* 02PA0MZ *and* 02PA3MZ *and* 02PA4MZ *and* 02PAXMZ) |
| Removal of whole system | 33233 *and* 33235 *without* (33217 *and* 33207 *and* 33208 *and* 33225 *and* 33206 *and* 33240 *and* 33249) | ( (0JPT0PZ *or* 0JPT3PZ) *and* (02PA0MZ *or* 02PA3MZ *or* 02PA4MZ *or* 02PAXMZ) ) *without* (02H63JZ *and* 02HK3JZ *and* 0JH606Z *and* 0JH636Z) |
| **ANY TRANSVENOUS PACEMAKER (CONVENTIONAL)** | | |
| Generator removal | 33233 *without* (33207 *and* 33234 *and* 33208 *and* 33206 *and* 33225 *and* 33240 *and* 33249 *and* 33235 *and* 33217 *and* 33216) | (0JPT0PZ *or* 0JPT3PZ) *without* (02H60JZ *and* 02H63JZ *and* 02H70JZ *and* 02H73JZ *and* 02HK0JZ *and* 02HK3JZ *and* 02PA0MZ *and* 02PA3MZ *and* 02PA4MZ *and* 02PAXMZ *and* 0JH604Z *and* 0JH605Z *and* 0JH606Z *and* 0JH636Z *and* 0JH607Z *and* 02H43JZ) |
| Lead removal | (33235 *or* 33234) *without* (33216 *and* 33217 *and* 33227 *and* 33228 *and* 33233) | (02PA0MZ *or* 02PA3MZ *or* 02PA4MZ *or* 02PAXMZ) without (0JH606Z *and* 0JH636Z *and* 02H63JZ *and* 02HK3JZ *and* 0JPT0PZ *and* 0JPT3PZ) |
| Insertion of lead only | (33216 *or* 33217) *without* (33234 *and* 33235) | (02H60JZ *or* 02H63JZ *or* 02H70JZ *or* 02H73JZ *or* 02HK0JZ *or* 02HK3JZ) *without* (0JH604Z *and* 0JH605Z *and* 0JH606Z *and* 0JH636Z *and* 0JPT0PZ *and* 0JPT3PZ *and* 02PA0MZ *and* 02PA3MZ *and* 02PA4MZ *and* 02PAXMZ) |
| Replacement of lead only | ( (33216 *or* 33217) *and* (33234 *or* 33235) ) without (33228 and 33227) | (02H60JZ *or* 02H63JZ *or* 02H70JZ *or* 02H73JZ *or* 02HK0JZ *or* 02HK3JZ) *and* (02PA0MZ *or* 02PA3MZ *or* 02PA4MZ *or* 02PAXMZ) *without* (0JPT0PZ and 0JPT3PZ) |
| Revision (e.g., repositioning generator or lead) | 33215, 33218, or 33222 | 02WA3MZ or 0JWT0PZ |
| **CRT-P with TRANSVENOUS RIGHT VENTRICULAR LEAD and CORONARY SINUS LEAD with or without RIGHT ATRIAL LEAD** | | |
| Insertion of whole CRT-P system | ( (33207 *or* 33208) *and* 33225 ) *without* (33233 *and* 33234) | (0JH607Z *and* 02HK3JZ *and* 02H43JZ) *without* 0JPT0PZ |
| Upgrade from single chamber pacemaker system to CRT-P, by adding a CS lead while using existing generator and existing RV lead | 33224 | 02H43JZ *without* (0JH607Z *and* 02HK3JZ *and* 0JPT0PZ) |
| Upgrade from single chamber pacemaker system to CRT-P, by removing old generator and replacing it with CRT-P generator, adding CS lead, and using existing RV lead - without an RA lead | ( (33228 *or* 33229) *and* 33225 ) *without* (33234 *and* 33235) | (0JH607Z *and* 0JPT0PZ *and* 02H43JZ) *without* (02HK3JZ *and* 02H63JZ) |
| Upgrade from single chamber pacemaker system to CRT-P, by removing old generator and replacing it with CRT-P generator, adding CS lead, adding RA lead, and using existing RV lead | 33206 *and* 33233 *and* 33225 | (0JH607Z *and* 0JPT0PZ *and* 02H43JZ *and* 02H63JZ) without 02HK3JZ |
| **CRT-D with TRANSVENOUS RIGHT VENTRICULAR LEAD and CORONARY SINUS LEAD with or without RIGHT ATRIAL LEAD** | | |
| Insertion of whole CRT-D system | (33249 *and* 33225) without (33233 *and* 33207 *and* 33208 *and* 33228 *and* 33229 *and* 33206 *and* 33240) | 0JH609Z *and* 02HK3KZ *and* 02H43KZ |
| Upgrade from single chamber pacemaker system to CRT-D, by removing old generator and replacing it with CRT-D generator, adding CS lead, and using existing RV lead - without an RA lead | (33240 *and* 33233 *and* 33225) *without* (33207 *and* 33234 *and* 33208 *and* 33206 *and* 33228 *and* 33229 *and* 33249) | 0JH609Z *and* 0JPT0PZ *and* 02H43KZ without (02HK3KZ *and* 02H63KZ) |
| Upgrade from single chamber pacemaker system to CRT-D, by removing old generator and replacing it with CRT-D generator, adding CS lead, adding RA lead, and using existing RV lead | (33249 *and* 33233 *and* 33225) *without* (33207 *and* 33234 *and* 33208 *and* 33206 *and* 33228 *and* 33229 *and* 33240) | 0JH609Z *and* 0JPT0PZ *and* 02H43KZ *and* 02H63KZ *without* 02HK3KZ |

^1^ For all measure definitions, codes can appear in any position on a claim and on all claim types (e.g. inpatient, outpatient, and carrier). ‘+’ signifies a wildcard and would include any alpha-numeric character in its place.

**Supplementary Table T2. ICD-10 Definitions of Patient Complications and Comorbidities**

| **Chronic Complication Measure** | **Definitions** |
| --- | --- |
| EMBOLISM AND THROMBOSIS | |
| THROMBOSIS DUE TO CARDIAC PROSTHETIC DEVICES, IMPLANTS AND GRAFTS | T82.867+ |
| EMBOLISM DUE TO CARDIAC PROSTHETIC DEVICES, IMPLANTS AND GRAFTS^3^ | T82.817+ |
| DEVICE-RELATED COMPLICATION | |
| MECHANICAL BREAKDOWN OF CARDIAC ELECTRONIC DEVICE | T82.11++ |
| DEVICE DISLODGEMENT OR DISPLACEMENT OF CARDIAC ELECTRONIC DEVICE | T82.12++ |
| OTHER MECHANICAL COMPLICATION OF CARDIAC ELECTRONIC DEVICE | T82.19++ |
| INFECTION AND INFLAMMATORY REACTION DUE TO OTHER CARDIAC AND VASCULAR DEVICES, IMPLANTS AND GRAFTS | T82.7+++ |
| HEMORRHAGE DUE TO CARDIAC PROSTHETIC DEVICE, IMPLANT AND GRAFTS | T82.837+ |
| PAIN DUE TO CARDIAC PROSTHETIC DEVICE, IMPLANT AND GRAFTS | T82.847+ |
| STENOSIS DUE TO CARDIAC PROSTHETIC DEVICE, IMPLANT AND GRAFTS | T82.857+ |
| POCKET COMPLICATION | T82.897+ |
| OTHER | |
| PERICARDITIS | I30.9 or I31.9 |
| HEMOTHORAX | (J95.62 or J95.831) plus J94.2  J95.72 plus J94.2 |
| **Patient Comorbidities** | **Definition** |
| ATRIAL FIBRILLATION | Any codes in the following ranges: I48.0-I48.2 or I48.91 |
| ATRIAL FLUTTER | Any codes in the following ranges: I48.3-I48.4 or I48.92 |
| CONGESTIVE HEART FAILURE | Any code in the following ranges: I09.81 or I11.0 or I13.0 or I13.2 or I50.20-I50.9 or I97.130-I97.131 |
| CHRONIC OBSTRUTIVE PULMONARY DISEASE | Any code in the following ranges: J43.0-J43.9 or J44.0-J44.9 or J47.0-J47.9 or J60-J63.6 |
| CHRONIC STEROID USE | Z79.52 |
| CORONARY ARTERY DISEASE | Any code in the following ranges: I25.10-I25.119 or I25.700-I25.739 or I25.790-I25.799 or I25.810 or I25.750-I75.769 or I25.811-I25.812 or I25.82-I25.84 |
| DIABETES | Any code in the following ranges: E08.00-E08.9 or E09.00-E09.9 or E10.10-E10.9 or E11.00-E11.9 or E13.00-E13.9) |
| HISTORY OF SUPRAVENTRICULAR TACHYCARDIA | I47.1 |
| HISTORY OF VENTRICULAR ARRYTHMIA | Any of the following codes: Z86.74 or I47.0 or I47.2 or I49.01 or I49.02 or I49.3 |
| HYPERLIPIDEMIA | E78.1-E78.5 |
| HYPERTENSION | Any code in the following ranges: I10 or I11.0-I11.9 or I12.0-I12.9 or I13.0-I13.2 or I15.0-I15.9 or I16.0-I16.9 or I97.3 |
| LEFT BUNDLE BRANCH BLOCK | I44.7 |
| PERIPHERAL VASCULAR DISEASE | Any code in the following ranges: I70.201-I70.299 or I70.301-I70.799 or I73.00-I73.9 |
| PRIOR CORONARY ARTERY BYPASS GRAFT | Any code in the following ranges: Z95.1 or T82.211A-T82.218S or I25.700-I25.739 or I25.790-I25.799 or I25.810 |
| PRIOR ACUTE MYOCARDIAL INFARCTION | Any code in the following ranges: I25.2 or I21.01-I21.4 |
| PRIOR PERCUTANEOUS CORONARY INTERVENTION | Any code in the following ranges: Z95.5 or Z98.61 or T82.855A-T82.855S |
| RENAL DYSFUNCTION | Any code in the following ranges: K76.7 or N17.0-N17.9 or N18.1-N18.9 or N19 or N28.9 or N99.0 or R39.2 |
| TRICUSPID VALVE DISEASE | Any code in the following ranges: I07.I-I07.9 or I08.1-I08.3 or I36.0-I36.9 or Q22.4 or Q22.8-Q22.9 |
| CONCOMITANT ATRIAL ABLATION | 93650 or 93653 or 93656 or 93657 or 02583ZZ + (I48.0-I48.2 or I48.91) |
| CONCOMITANT TRANSCATHETER AORTIC VALVE REPLACEMENT | 33361or 33362 or 33363 or 33364 or 33365 or 33366 or 02RF38Z or 02RF38H |
| PRIOR TRANSCATHETER AORTIC VALVE REPLACEMENT | 33361or 33362 or 33363 or 33364 or 33365 or 33366 or 02RF38Z or 02RF38H |
| COVID-19 | U071, B9729 |
| USE OF TEMPORARY PACING DURING THE IMPLANT HOSPITALIZATION | 33210, 33211, 5A1213Z, 5A1223Z |
| USE OF LIFESAVING PROCEDURES DURING THE IMPLANT HOSPITALIZATION | 92920, 5A12012, 5A19054, 33946, 33947, 33952, 33954, 33956, 33958, 33962, 33964, 33987, 33988, 5A1522H, 5A1522G, 5A1522F, 02H703Z, 02HL03Z, 03U30JZ, 03U40JZ, 03UK0JZ, 03UL0JZ, 03UM0JZ, 03UN0JZ, 03WY0YZ, 05WY0YZ, 06WY0YZ, 99291, 99292, 94002, 94003, 5A0920Z, 5A09357, 5A09358, 5A09359, 5A0935A, 5A0935B, 5A0935Z, 5A09457, 5A09458, 5A09459, 5A0945A, 5A0945B, 5A0945Z, 5A09557, 5A09558, 5A09559, 5A0955B, 5A0955Z |

**Supplementary Table T3. Unadjusted event rates of chronic complications, device-related reinterventions, and all-cause mortality at 2 years**

|  | **Micra AV (N=7,552)** | **DC-TV (N=110,558)** |
| --- | --- | --- |
| **Overall complications** | **397 (5.3%)** | **8,958 (8.1%)** |
| Embolism and Thrombosis | 17 (0.2%) | 171 (0.2%) |
| Device-related complications | 228 (3.0%) | 7,118 (6.4%) |
| Breakdown | 127 (1.7%) | 2,807 (2.5%) |
| Dislodgement | 36 (0.5%) | 2,936 (2.7%) |
| Other mechanical failure | 55 (0.7%) | 1,437 (1.3%) |
| Infection | * | 471 (0.4%) |
| Device pain | * | 406 (0.4%) |
| Device stenosis | 36 (0.5%) | 410 (0.4%) |
| Pocket complications | N/A | 1,465 (1.3%) |
| Other complications | 167 (2.2%) | 1,931 (1.8%) |
| Pericarditis | 120 (1.6%) | 1,603 (1.5%) |
| Hemothorax | 54 (0.7%) | 503 (0.5%) |
| **Overall reinterventions** | **232 (3.1%)** | **5,247 (4.8%)** |
| Revisions | * | 1,635 (1.5%) |
| Lead-related reinterventions | N/A | 1270 (1.2%) |
| Replacement | 35 (0.5%) | 701 (0.6%) |
| System switch^1^ | 85 (1.1%) | 130 (0.1%) |
| Removal | * | 574 (0.5%) |
| Upgrade to CRT | 97 (1.3%) | 1,364 (1.2%) |
| **All-cause mortality** | **2,363 (31.3%)** | **16,202 (14.7%)** |

^1^ Replacement with the opposite type of device. *****Cell value between 1 and 10. DC-TV: Dual-Chamber Transvenous pacemaker. N/A: Not applicable. NE: Not estimable. CRT: Cardiac Resynchronization Therapy device.

**Supplementary Table T4. Results from falsification test – hip fracture outcome**

|  | **Micra AV**  **(N=7,552)** | **Dual-Chamber Transvenous (N=110,558)** | **Micra AV vs. Dual-Chamber Transvenous** | | | |
| --- | --- | --- | --- | --- | --- | --- |
|  | **Observed Events (%)** | **Observed Events (%)** | **Unadjusted HR (95% CI)** | **P-Value** | **Adjusted HR**  **(95% CI)** | **P-Value** |
|  |  |  |  |  |  |  |
| **Hip Fracture^1^** | 103 (1.4%) | 928 (0.8%) | 1.653 (1.348 - 2.027) | <.0001 | 1.275 (1.028 - 1.581) | 0.0273 |

^1^ Hip fracture identified with ICD-10 diagnosis codes S72.0XXX, S72.1XXX, S72.2XXX.

**Supplementary Table T5. All-cause mortality subgroup analyses – AVB patients only**

|  | **Micra AV**  **(N=5,607)** | **Dual-Chamber Transvenous (N=52,652)** | **Micra AV vs. Dual-Chamber Transvenous** | | | |
| --- | --- | --- | --- | --- | --- | --- |
|  | **Observed Events (%)** | **Observed Events (%)** | **Unadjusted HR (95% CI)** | **P-Value** | **Adjusted HR**  **(95% CI)** | **P-Value** |
|  |  |  |  |  |  |  |
| **All-cause mortality** | 1,770 (31.6%) | 8,560 (16.3%) | 2.240 (2.108 - 2.381) | <.0001 | 1.509 (1.414 - 1.611) | <.0001 |
| **Conditional all-cause mortality^1^** | 832 (18.4%) | 4,949 (10.4%) | 1.942 (1.792 - 2.104) | <.0001 | 1.378 (1.266 - 1.499) | <.0001 |

^1^ Sample size is Micra AV N=4,518 and DC Transvenous N=47,778.

**Appendix Figure F1. CONSORT Diagram
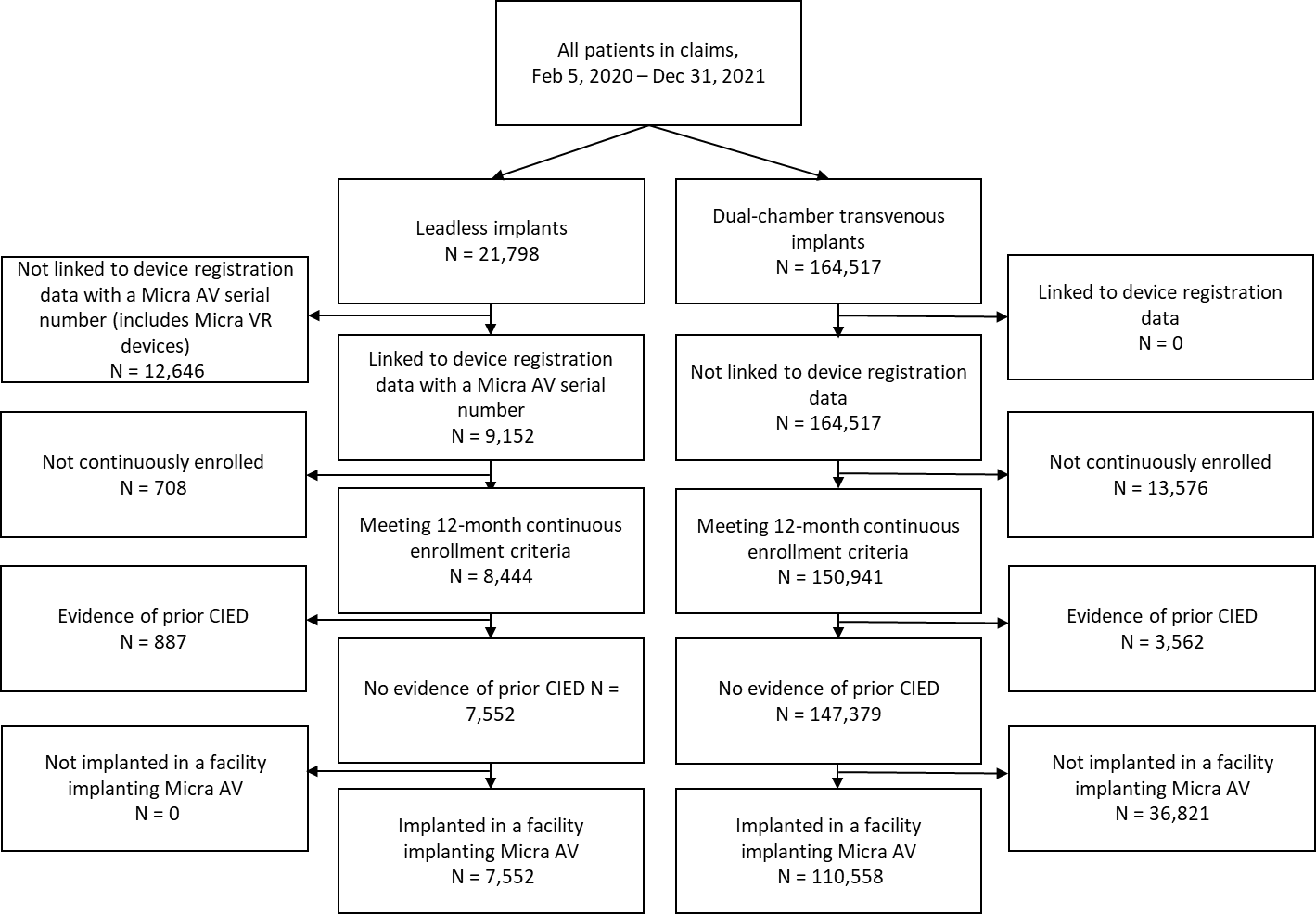
**

**Supplementary Figure F2.** **Unadjusted Cumulative Incidence Functions of chronic complications and device-related reinterventions at 2 years in Micra AV vs Dual-Chamber Transvenous patients.**

Unadjusted Hazard Ratios (HR), 95% Confidence Intervals (CI), and cumulative incidence functions for 2-year chronic complications (Panel A) and device-related reinterventions (Panel B) based on Fine-Gray competing risk models.

**Supplementary Figure F3. Cumulative Incidence Functions of conditional all-cause mortality at 2 years in Micra AV vs Dual-Chamber Transvenous patients.**

Unadjusted (Panel A) and adjusted (Panel B) Hazard Ratios (HR), 95% Confidence Intervals (CI), and cumulative incidence functions for 2-year conditional all-cause mortality based on Fine-Gray competing risk models.
